# Data-driven water quality prediction for wastewater treatment plants

**DOI:** 10.1016/j.heliyon.2024.e36940

**Published:** 2024-08-28

**Authors:** Haitham Abdulmohsin Afan, Wan Hanna Melini Wan Mohtar, Faidhalrahman Khaleel, Ammar Hatem Kamel, Saif Saad Mansoor, Riyadh Alsultani, Ali Najah Ahmed, Mohsen Sherif, Ahmed El-Shafie

**Affiliations:** a Upper Euphrates Basin Developing Center, University of Anbar, Iraq; b Department of Civil Engineering, Faculty of Engineering and Built Environment, Universiti Kebangsaan Malaysia, 43600, UKM Bangi, Selangor, Malaysia; c Environmental Management Center, Institute of Climate Change, Universiti Kebangsaan Malaysia, 43600, UKM Bangi, Selangor, Malaysia; d Ministry of Electricity, The State Company of Electricity Production GCEP Middle Region, Baghdad, Iraq; e Building and Construction Techniques Engineering Department, College of Engineering and Engineering Techniques, Al-Mustaqbal University, 51001, Babylon, Iraq; f Research Centre For Human-Machine Collaboration (HUMAC), School of Engineering and Technology, Sunway University, No. 5, Jalan Universiti, Bandar Sunway, 47500, Selangor Darul Ehsan, Malaysia; g Department of Engineering, School of Engineering and Technology, Sunway University, No. 5, Jalan Universiti, Bandar Sunway, 47500, Selangor Darul Ehsan, Malaysia; h National Water and Energy Center, United Arab Emirate University, P.O. Box 15551, Al Ain, United Arab Emirates; i Civil and Environmental Eng. Dept., College of Engineering, United Arab Emirates University, Al Ain, 15551, United Arab Emirates; j Department of Civil Engineering, Atatürk University, 25240, Erzurum, Turkey; k Dams and Water Resources Department, College of Engineering, University of Anbar, Iraq

**Keywords:** Water quality prediction, Machine learning, Wastewater treatment plants, Neural networks

## Abstract

Monitoring and managing wastewater treatment plants (WWTPs) is crucial for environmental protection. The presection of the quality of treated water is essential for energy efficient operation. The current research presents a comprehensive comparison of machine learning models for water quality parameter prediction in WWTPs. Four machine learning models presented in MLP, GFFR, MLP-PCA, and RBF were employed in this study. The primary notion of this study is to apply the proposed models using two distinct modeling scenarios. The first scenario represents a straightforward approach by utilizing the inputs and outputs of WWTPs; meanwhile, the second scenario involves using multi-step modeling techniques, which incorporate intermediate outputs induced by primary and secondary settlers. The study also investigates the potential of the adopted models to handle high dimensional data as a result of the multi-step modeling since more data points and outputs are progressively integrated at each step. The results show that the GFFR model outperforms the other models across both scenarios, specifically in the second scenario in predicting conductivity (COND) by providing higher correlation accuracy (R = 0.893) and lower prediction deviations (NRMSE = 0.091 and NMAE = 0.071). However, all models across both scenarios struggle to predict the other water quality parameters, generating significantly lower prediction correlations and higher prediction deviations. Nonetheless, the innovative multi-step technique in scenario two has significantly boosted the prediction capacity of all models, with improvement ranging from 0.2 % to 157 % and an average of 60 %. The implementation of AI models has proven its ability to accomplish high accuracy for WQ parameter prediction, highlighting the impact of leveraging intermediate process data.

## 1 Introduction

The growing concerns over environmental issues have led experts to focus on the proper functioning and management of wastewater treatment plants (WWTPs) [[1], [2], [3]]. Worldwide, over 80 % of swage (undertreated or without treatment) are being discharged or shunted [4]. Consequently, better water treatment methods can significantly benefit humanity and the environment. Currently, many methods, such as physical, chemical, and biological, have been adopted for wastewater treatment. Among these methods, the biological method has made considerable strides in wastewater treatment and has become the most common method due to its efficiency and cost-effectiveness [5]. However, WWTPs are considered a very complex nonlinear system, and it is not easy to measure quality indicators such as biochemical oxygen demand (BOD), chemical oxygen demand (COD), and suspended solids (SS) since the measurement is sensitive to disturbance, working conditions, and environment. Consequently, the values of quality indicators at the inlet of WWTPs, and the quantity of wastewater flowing into WWTPs vary widely, which has a significant effect on wastewater treatment at the biological reactors. Therefore, the biological reactor settings need to be continuously adjusted to ensure the high operational reliability of the treatment plant and to reach the required wastewater quality indicators at the outfall. In this regard, a massive amount of data collected by the WWTPs can be incorporated to develop and enhance the operation of treatment plants by forecasting and simulating the process of wastewater treatment. Predicting approaches can be classified generally into two categories: first principle and data-driven models based on constructed models’ characteristics [6].

In WWTPs, the most commonly used first principle-based model is the activated sludge model (ASM) [7], introduced by the International Water Association (IWA). In the ASM model, a suspension of bacterial biomass acts as a sorbent to remove the pollutants. An activated sludged WWTPs can eliminate organic carbonaceous materials as well as phosphorus (P) and biological nitrogen (N). Furthermore, several different configurations of activated sludge processes have evolved in the last decades [8]. In order to have a better understanding of nitrite dynamics, particularly in communal wastewater treatment, D. Kaelin et al. [9] proposed an extended version of activated sludge model No.3 (ASM3) to forecast the nitrogen content. Yang et al. [10] introduced a fully coupled ASM model (FCASM) to determine the effluent ammonia-nitrogen (NH4−N), taking into consideration specific operating parameters. However, utilizing ASM for real-time applications presents significant challenges. For instance, the characterization of the organic matter and determining some characteristics of volatile fatty acid (VFA) is rather challenging, time-consuming, and costly but essential for the calibration process [11]. Moreover, these models suffer when it comes to high uncertainties, nonlinearities, and the variation of environmental conditions, which are the main characteristics of WWTPs [12].

On the other hand, data-driven models use input and output data to create an equivalent model that is independent of the process mechanism. In other words, data-driven models do not require a thorough understanding of the process’ mechanism in the existence of enough data. Over the last decades, data-driven models have been implemented to tackle a wide range of complex problems in general [[13], [14], [15], [16], [17], [18], [19], [20], [21], [22]] and to predict WWTPs quality parameters in particular [[23], [24], [25], [26], [27], [28], [29], [30], [31], [32], [33], [34], [35], [36]]. H. Guo et al. [26] compared the performance of the artificial neural network (ANN) and support vector machine (SVM) in total nitrogen concentration (T−N) prediction for a one-day interval. The results showed that the ANN model provides better performance than the SVM model. Li et al. [37] introduced a self-organizing cascade neural network (SCNN) with random weights to capture the underlying nonlinearity in wastewater treatment. The results showed that the SCNN model provides a good prediction accuracy on BOD and total phosphorus (TP). Zhu et al. [38] proposed a hybrid model that integrates multiple linear regression (MLR) and ANN to predict BOD concentrations. Meng et al. [39] introduced the adaptive task-oriented radial basis function network (ATO−RBF) to predict effluent BOD and effluent T−N. The result showed that the ATO−RBF model provides superior predictions compared to the conventional approaches. Y. Zhang et al. [40] introduced a deep learning approach presented in the long short-term memory model (LSTM) coupled with global sensitivity analysis, which is based on Shapley additive explanations (SHAP) to predict water quality key indicators such as chemical oxygen demand (COD), total nitrogen and phosphorous. The findings showed the effectiveness of the LSTM model in addressing the limitations of the traditional approach by adopting deep learning approaches.

Despite the robustness of these models, the existing models are broadly focused on single-step prediction by exclusively utilizing inputs and outputs of the WWTPs without involving intermediate steps. Consequently, the models may miss valuable information that is provided by the intermediate stage of water treatment, such as primary and secondary settler, influencing the model's adaptability and practicality.

Given these facts, this study is conducted to address these gaps and to investigate the potential of various machine learning models in predicting water quality parameters. Furthermore, it investigates their adaptability to predict these parameters utilizing two different modeling scenarios: single-step and multiple-step. The single-step is a straightforward scenario that exclusively utilizes WWTP inputs and outputs. On the other hand, the multi-step scenario utilizes intermediate stages, such as primary and secondary settler outputs, in the development process of the adopted model to reach the final prediction. Furthermore, the adopted technique gives significant insight into the performance of adopted models against high-dimension data points induced by the progressive generation of new data at each step, setting a new benchmark in the predicting process of WWTPs process.

## 2 Data acquisition

For the developing process of the proposed models, the data were obtained online from Ref. [41], which represents the daily measures of sensors in urban WWTP. The WWTP process is categorized into four groups: WWTP input, primary settler, secondary settler, and WWTP output. The data are obtained for each group. For the WWTP input data, such as flow (Q-E), Zinc (ZN-E), PH (PH-E), biological oxygen demand (BOD-E), chemical oxygen demand (COD-E), suspended solid (SS-E), volatile suspended solids (SSV-E), sediments (SED-E), and conductivity (COND-E) is obtained. For the primary settler, PH (PH-P), biological oxygen demand (BOD-P), suspended solid (SS-P), volatile suspended solids (SSV-P), sediments (SED-P), and conductivity (COND-P) data are obtained. For the secondary settler, PH (PH-D), biological oxygen demand (BOD-D), chemical oxygen demand (COD-D), suspended solid (SS-D), volatile suspended solids (SSV-D), sediments (SED-D), and conductivity (COND-D) data are obtained. Finally, for the WWTP output, data such as PH (PH-S), biological oxygen demand (BOD-S), chemical oxygen demand (COD-S), suspended solid (SS-S), volatile suspended solids (SSV-S), sediments (SED-S), and conductivity (COND-S) data are obtained. The data are divided into three parts: training, which represents 70 % of the data; validation, which represents 15 % of the data; and testing, which represents the remaining 15 % of the data. The statistical characteristics of the data are depicted in Table 1.

**Table 1:** Statistical descriptions of the obtained data.

Parameter/statistical matrices	Minimum	Maximum	Mean	Standard deviation
WWTP INPUT				
Q-E	10000	60081	37226.56	6571.46
ZN-E	0.1	33.5	2.36	2.74
PH-E	6.9	8.7	7.81	0.24
BOD-E	31	438	188.71	60.69
COD-E	81	941	406.89	119.67
SS-E	98	2008	227.44	135.81
SSV-E	13.2	85	61.39	12.28
SED-E	0.4	36	4.59	2.67
COND-E	651	3230	1478.62	394.89
PRIMARY SETTLER				
PH-P	7.3	8.5	7.83	0.22
BOD-P	32	517	206.2	71.92
SS-P	104	1692	253.95	147.45
SSV-P	7.1	93.5	60.37	12.26
SED-P	1	46	5.03	3.27
COND-P	646	3170	1496.03	402.58
SECONDARY SETTLER				
PH-D	7.1	8.4	7.81	0.19
BOD-D	26	285	122.34	36.02
COD-D	80	511	274.04	73.48
SS-D	49	244	94.22	23.94
SSV-D	20.2	100	72.96	10.34
SED-D	0	3.5	0.41	0.37
COND-D	85	3690	1490.56	399.99
WWTP OUTPUT				
PH-S	7	9.7	7.7	0.18
BOD-S	3	320	19.98	17.2
COD-S	9	350	87.29	38.35
SS-S	6	238	22.23	16.25
SSV-S	29.2	100	80.15	9
SED-S	0	3.5	0.03	0.19
COND-S	683	3950	1494.81	387.53

The missing values were addressed through multiple imputation by chained equations (MICE) in this study, this imputation technique involves imputing a missing value depending on the other observable variables in a dataset. This method produced more than one complete data set which provided for valid statistical inferences because it made adjustments for imprecision which was an attribute of the imputing process. Both datasets were analyzed separately, and in the final analyses, results obtained from the separate datasets were combined, and the measure of variability between imputations was taken using Rubin's rules.

## 3 Model development

**Fig. 1:**
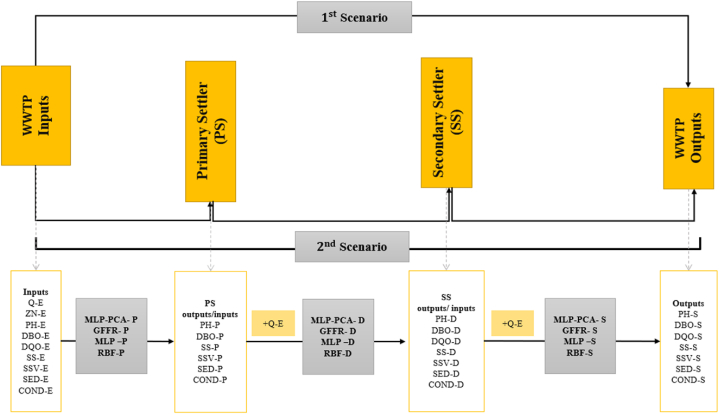
Establishing models process.

In order to predict water quality parameters, four models, namely MLP−PCA, GFFR, MLP, and RBF, have been established. The prediction process was done using two different scenarios, as shown in Fig. 1, to select the most efficient path that achieves the desired prediction. In the first scenario, the proposed models are trained, validated, and tested using input and output parameters of the wastewater treatment plant only without considering both primary and secondary settler outputs in the model development process. While in the second scenario, a multi-step process was conducted before reaching the final outputs. Furthermore, since the discharge (Q−E) in the wastewater treatment system does not change, it has been considered as additional input in each step of scenario two. The multi-step process can be presented in the following order:

1. Establishing the proposed models using wastewater treatment plant inputs and primary settler outputs.
2. After establishing models, new data are generated using the same models and used as inputs, followed by the discharge to the next step.
3. Establishing the proposed models using the generated input (from step two) and secondary settler outputs.
4. After establishing models from step three, new data are generated using the same models and used as inputs along with the discharge to the final step.
5. Establishing the proposed models using the generated inputs (step four) and wastewater treatment plant outputs.

The performance of the proposed models in both scenarios is evaluated in the training and testing phases using multiple statistical matrices and graphical appraisals.

### 3.1 Radial basis function neural network

The radial basis function (RBF) is considered an FFNN, and its structure is similar to MLP. RBF structure consists of three layers: input, hidden, and output. The main difference between RBF and MLP is that RBF contains only one hidden layer [[42], [43], [44]]. In addition, the training process is done within one stage instead of performing an iterative process as with MLP. In the input layer, the input parameters are received, then passed these parameters to the hidden layer, which has a radial basis function as an activation function. The Gaussian function is considered the most common among different variants of radial basis functions. In the output layer, the outputs of the hidden layer are summed, as illustrated in Equation (1).

(1) $$\gamma =\sum_{r=1}^{l}w_{r}\sigma_{r}$$

Where w is the weight, $\sigma_{r}$ are the neuron outputs of the hidden layer and can be mathematically expressed in Equation (2).

(2) $$\sigma_{r}=\exp\left(-\frac{{\left(x_{r}-c_{r}\right)}^{2}}{\beta_{r}^{2}}\right)$$

Where x is the input vector, $\beta_{r}$ is the Gaussian function spread and $c_{r}$ is the basis function.

### 3.2 Multi-layer perceptron

**Fig. 2:**
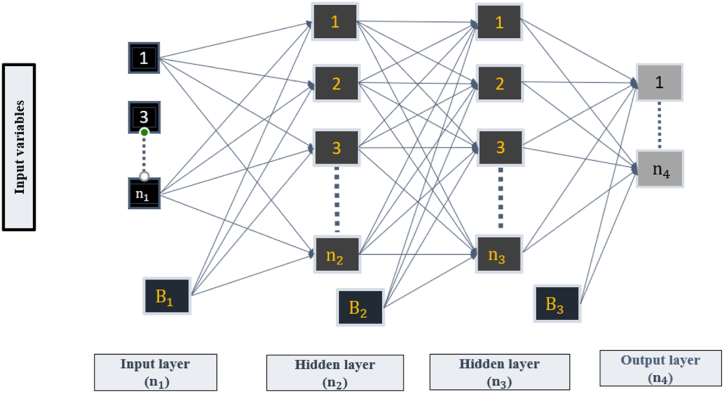
The structure of MLP.

Multi-layer perceptron (MLP) is considered the most dominating network in ANN due to its exceptional learning ability, enabling it to learn deeper connections among data and thus providing a more effective and powerful tool for prediction and classification tasks. Furthermore, MLP can address different issues by the standard ANN, such as the shallow layers and more straightforward structure, making the latter stuck in the local minima and generating overfitting predictions. The structure of MLP consists of an input layer, a hidden layer (s), and an output layer. Input data is received by the input layer, which transfers them to the hidden layer (s) where the features are processed. The output layer is utilized to reveal the predicted results. Fig. 2 shows the main structure of MLP. Moreover, each layer is made of several neurons, which are connected between layers using weight (w) and bias (B). The output of the neuron (n) in the hidden layer is calculated using the following equation.

(3) $$H_{n}=\sigma_{1}\left(\sum_{r=1}^{R}w_{\text{nr}}X_{r}+B_{r}\right)$$

$w_{\text{nr}}$ and $B_{r}$ are the hidden layer's weights and biases and $\sigma_{1}\left(.\right)$ is the activation function. The output (Y) of the network is illustrated in Equation (2).

(4) $$Y=\sigma_{2}\left(\sum_{j=1}^{L}w_{\text{kj}}H_{i}+B_{O}\right)$$

Where $w_{\text{kj}}$, $B_{O}$ are weights and biases, respectively. $\sigma_{2}\left(.\right)$ is the activation function of the output layer.

### 3.3 Generalized regression neural network

**Fig. 3:**
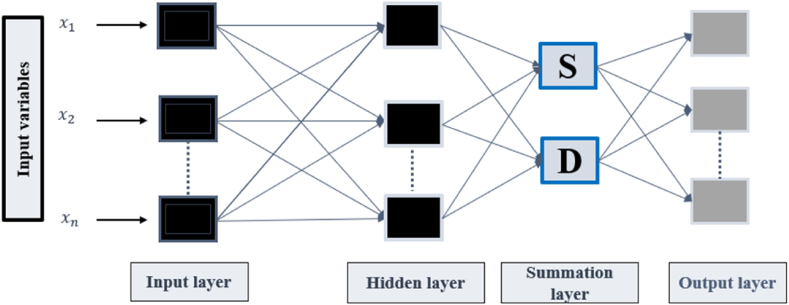
The structure of GRNN.

Generalized regression neural network (GRNN) is considered a variant of RBF introduced by Specht [45] to perform classification, regression, and classification tasks. As shown in Fig. 3, the structure of GRNN involves four main layers: input, hidden, summation, and output. The input layer, where the data are received, includes neurons equal to the input vector's dimensions. The radial base layer is presented in the hidden layer with neurons equal to the training samples. In this case, the basis function is the Gaussian function $\left(G\left(x,x_{m}\right)\right)$ and the $m^{th}$ neuron center vector is $x_{m}$. The summation layer involves two types of neurons, namely the denominator unit and the molecular unit. The denominator unit (Equation (5)) calculates the hidden layer neurons' algebraic sum, while the molecular unit (Equation (6)) calculates the summed weights of the hidden layer neurons.

(5) $$S=\sum_{m=1}^{M}\omega_{m}\;\exp\;\left[-G\left(x,x_{m}\right)\right]$$

(6) $$D=\sum_{m=1}^{M}\;\exp\;\left[-G\left(x,x_{m}\right)\right]$$

In the output layer, the output value γ is estimated by dividing the denominator unit by the molecular unit, as shown in Equation (7).

(7) $$Y\left(X\right)=\frac{\sum_{m=1}^{M}\omega_{r}\;\exp\;\left[-G\left(x,x_{m}\right)\right]}{\sum_{m=1}^{M}\;\exp\;\left[-G\left(x,x_{m}\right)\right]\;}$$

### 3.4 Principle components analysis

Principle components analysis (PCA) is a technique for constructing new variables that are linear composites of the original variables. Furthermore, these new variables are uncorrelated and have the same maximum number of variables as the original ones. In other words, PCA searches for the projection that best describes the data in terms of least-square. A set of n predictor variables may be denoted mathematically as:

(8) $$m_{i}={\left(m_{i}\left(1\right),m_{i}\left(2\right),\cdots ,m_{i}\left(p\right)\right)}^{T};\;i=1,2,\cdots ,r$$

The covariance matrix of the sample is given as follows:

(9) $$M=\frac{1}{r}\sum_{i=1}^{r}m_{i}.{m_{i}}^{T}$$

In PCA, the transformation of the predictor variables to new variables is as follows:

(10) $$\gamma_{i}=U^{T}m_{i}$$

Where U is N×N orthogonal matrix. The sample covariance matrix's $j^{th}$ Eigenvector corsponds to the $j^{th}$ column of the U matrix. The following equation is used to solve it.

(11) $$\delta_{j}v_{j}=Mv_{j},j=1,2,\cdots ,N$$

Where $\delta_{j}$ and $v_{j}$ are the Eigenvalue and corresponding Eigenvector of M, respectively. Equation (1) is used to determine the orthogonal fraction of the predictor variable $v_{i}$ after transforming $m_{i}$. The resultant component is designated as the principle component.

## 4 Results and discussion

### 4.1 Scenario 1

**Fig. 4:**
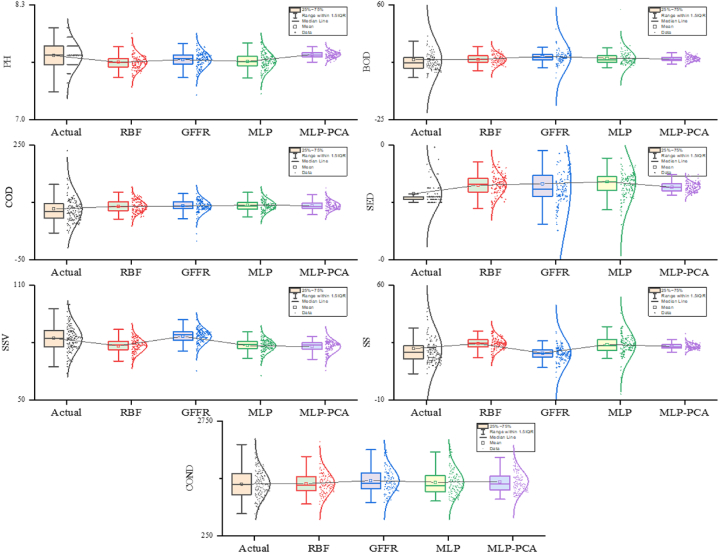
Boxplot showing the performance of the proposed models: Testing phase.

The proposed models are developed using water treatment plant inputs and outputs in this scenario (single-step). The performance of the proposed models in the training phase is presented in Table 2, showing that all models performed poorly in predicting water-quality parameters (except COND-S) by providing high margins of error and less prediction accuracy. Meanwhile, the proposed models provide a moderate performance in predicting the conductivity (COND−S), with the GFFR model taking the lead with higher prediction accuracy (R=0.897), indicating that the model explains 89.7 % of the variance in data and lower margins of error (NRMSE=0.06,NMAE=0.042) between actual and predicted value compared to the other models. Table 3 shows the performance of the proposed models during the testing phase. According to Table 3, all models provide significantly poor performance in predicting all water quality parameters except the COND−S parameter, where the proposed models show a moderate performance in predicting the latter, reaching the best performance with the GFFR model with lower prediction errors (NRMSE=0.05,NMAE=0.036) between actual and predicted values and higher prediction capacity (R=0.891), where the model can explain 89.1 % of variance in data compared to the other models. Fig. 4 presents a box plot showing the distribution of the predictive values for each model compared to the actual one. The GFFR, MLP, and MLP-PCA models show prediction distributions that are close to the actual ones with medians that are closely aligned. Furthermore, these models have smaller interquartile range (IQR) values, indicating the strong performance and reliability of models in predicting conductivity. However, with regard to other parameters, all models showed significant deviations and outliers from the actual ones, suggesting the limitations of these models in handling complex relationships and patterns.

**Table 2:** The performance of the proposed models through the training phase: First scenario.

Matrices/Parameters	PH-S	BOD-S	COD-S	SS-S	SSV-S	SED-S	COND-S
MLP–PCA							
NRMSE	0.156	0.063	0.109	0.082	0.122	**0.069**	0.072
NMAE	0.126	**0.022**	0.071	**0.041**	0.091	**0.018**	0.049
R	−0.055	0.123	0.331	0.126	0.382	0.104	0.848
GFFR							
NRMSE	0.136	0.063	0.108	0.086	0.137	0.071	**0.060**
NMAE	0.109	0.025	0.074	0.042	0.104	**0.018**	**0.042**
R	0.477	0.124	0.388	0.164	**0.477**	0.075	**0.897**
MLP							
NRMSE	**0.127**	0.063	**0.105**	0.081	**0.116**	0.070	0.060
NMAE	**0.104**	0.024	**0.067**	0.043	**0.087**	0.020	0.042
R	**0.539**	0.176	**0.418**	0.182	**0.477**	0.090	0.894
RBF							
NRMSE	0.130	**0.062**	0.106	**0.080**	0.118	**0.069**	0.072
NMAE	0.105	0.023	0.068	0.042	**0.087**	0.019	0.050
R	0.514	**0.226**	0.403	**0.229**	0.445	**0.158**	0.841

**Table 3:** The performance of the proposed models through the testing phase: First scenario.

Matrices/Parameters	PH-S	BOD-S	COD-S	SS-S	SSV-S	SED-S	COND-S
MLP−PCA							
NRMSE	**0.115**	0.044	0.121	0.054	0.124	0.018	0.058
NMAE	**0.086**	0.022	0.085	0.033	0.098	0.013	0.046
R	**0.284**	0.082	0.225	0.047	0.121	−0.079	0.823
GFFR							
NRMSE	0.134	0.055	0.119	0.068	**0.107**	0.051	**0.050**
NMAE	0.094	0.029	0.083	0.037	**0.083**	0.024	**0.036**
R	0.189	0.066	0.291	0.001	**0.202**	−0.025	**0.891**
MLP							
NRMSE	0.139	0.046	**0.117**	0.065	0.116	**0.029**	0.056
NMAE	0.107	0.024	**0.083**	0.038	0.093	**0.020**	0.036
R	0.219	0.118	**0.349**	0.086	0.218	**0.099**	0.836
RBF							
NRMSE	0.134	**0.044**	0.121	**0.057**	0.122	0.021	0.081
NMAE	0.104	**0.022**	0.085	**0.040**	0.096	0.016	0.052
R	0.287	**0.153**	0.234	**0.108**	0.142	0.002	0.601

The results from the first scenario highlight the limitation of the single-step modeling technique in predicting most WWTP quality parameters, suggesting the shortcomings of the models in capturing the complex, highly nonlinear relationships within the process of WWTPs as a result of solely relying on input and outputs without considering the intermediate steps. The superiority of the GFFR model in predicting CONS-S relies upon the model's capacity to capture nonlinear and complex relationships while it still falls short in a boarder context.

### 4.2 Scenario 2

As mentioned earlier, this scenario involves a multi-step process to reach the prediction of the final water quality parameters. The first step is between ${\text{WWTP}}^{\prime }$ s input parameters and primary settler outputs, and the performance of the proposed models during this step is presented in Table 4, Table 5. Table 4 shows the performance of the proposed models during the training phase, showing that the GFFR gives the best performance in terms of suspended solids (SS−P), volatile suspended solids (SSV−P), sediments (SED−P), and conductivity (COND−P). Furthermore, the performance of GFFR in COND−P parameter prediction is significantly higher with R=0.969, indicating 96.6 % of the variance in data is explained by the model, and lower prediction deviations with NRMSE=0.044 and NMAE=0.022 compared to other parameters. Similarly, the GFFR showed superior performance in predicting suspended solids (SS−P), volatile suspended solids (SSV−P), and sediments (SED−P), demonstrating a strong correlation and prediction capacity (R = 0894, R = 0.898, and R = 0.824, respectively) and lower deviations between actual and predicted values. Meanwhile, the MLP mode shows exceptional performance in predicting PH−P with R=0.892, indicating 89.2 % of data variance is explained by the model and lower prediction deviations with NRMSE=0.088 and NMAE=0.067. Additionally, the model performs well in predicting SS−P, SSV−P, and COND−P, indicating the model's ability to deal with high nonlinear relationships. However, both models show significantly poor performance regarding biological demand of oxygen (BOD−P) prediction.

**Table 4:** The performance of the proposed models during the training phase: First step.

Matrices/Parameters	PH-P	BOD-P	SS-P	SSV-P	SED-P	COND-P
MLP−PCA						
NRMSE	0.107	0.113	0.104	0.148	0.077	0.164
NMAE	0.085	0.085	0.060	0.115	0.047	0.128
R	0.861	0.678	0.186	0.405	0.359	0.355
GFFR						
NRMSE	0.109	0.111	**0.048**	**0.072**	**0.049**	**0.044**
NMAE	0.088	0.084	**0.031**	**0.054**	**0.034**	**0.026**
R	**0.895**	0.696	**0.894**	**0.898**	**0.824**	**0.969**
MLP						
NRMSE	**0.088**	**0.109**	0.056	0.072	0.053	0.045
NMAE	**0.067**	**0.080**	0.036	0.054	0.036	0.027
R	0.892	**0.702**	0.849	0.896	0.770	0.967
RBF						
NRMSE	0.105	0.111	0.059	0.077	0.055	0.060
NMAE	0.083	0.082	0.039	0.059	0.038	0.040
R	0.843	0.692	0.828	0.880	0.750	0.940

**Table 5:** The performance of the proposed models during the testing phase: First step.

Matrices/Parameters	PH-P	BOD-P	SS-P	SSV-P	SED-P	COND-P
MLP−PCA						
NRMSE	0.106	0.102	0.159	0.135	0.135	0.136
NMAE	0.088	0.084	0.078	0.103	0.063	0.113
R	0.834	0.718	0.036	0.040	0.100	0.197
GFFR						
NRMSE	0.098	0.106	0.069	0.080	0.078	0.034
NMAE	0.081	0.090	0.040	0.060	0.043	0.023
R	0.856	0.724	0.924	0.803	0.822	0.967
MLP						
NRMSE	**0.074**	**0.096**	**0.060**	**0.079**	**0.073**	**0.027**
NMAE	**0.058**	**0.076**	**0.037**	**0.060**	**0.038**	**0.020**
R	**0.869**	**0.728**	**0.927**	**0.808**	**0.861**	**0.979**
RBF						
NRMSE	0.101	0.098	0.074	0.086	0.095	0.067
NMAE	0.077	0.078	0.042	0.068	0.047	0.042
R	0.736	0.717	0.899	0.771	0.713	0.857

On the other hand, during the testing phase, the MLP model gives higher performance for all parameters, reaching an excellent prediction capacity in COND−P prediction with R=0.979, NRMSE=0.027, and NMAE=0.02, followed by SS−P with R=0.927, NRMSE=0.06, and NMAE=0.037, indicating superior predicting capacity, where the model explains the majority of variance in data, and minimal errors induced. Meanwhile, the GFFR model continues to demonstrate high performance, particularly for SS−P and COND−P, with correlation values of 0.924 and 0.967, respectively, proving model stability and reliability. The RBF model showed a moderate performance in predicting WWTP parameters. Meanwhile, the MLP-PCA showed a good performance in PH−P prediction with a high correlation coefficient (R=0.861) and lower prediction deviations in terms of NRMSE and NMAE. However, the model struggles with other parameters, indicating model inconsistency.

Moving to the second step, which involves using the previous models to generate new values for the parameters (PH−P,SS−P,SSV−P,SED−P,andCOND−P) and using them as input along with the discharge (Q−E) to develop new models taking into account the outputs of the secondary settler for the developing process. The performance of the proposed models in this step is presented in Table 6, Table 7, which represent the performance during the training and testing phases, respectively. During the training phase, both MLP and GFFR models showed slightly similar performance. The MLP provides a good prediction capacity regarding COND−D with R=0.945, NRMSE=0.037, and NMAE=0.024, suggesting that the model can capture the relationship between input parameters and conductivity. Similarly, the MLP shows a moderate performance in terms of PH−D with R=0.81, NRMSE=0.093, and NMAE=0.072, indicating a reasonable ability to predict PH values. However, the MLP model shows significantly poor performance regarding the other parameters, such as (BOD−P), (COD−P), (SS−P), (SSV−P), and (SED−P), with R values ranging from 0.364 to 0.725, indicating the lack of the generalization ability of the model across different WWTP parameters. The GFFR model shows a similar trend with superior performance in predicting COND−D with R=0.947, NRMSE=0.039, NMAE=0.028, and moderate performance with PH−D values. However, the model struggles with regard to the other parameters, suggesting that the model has a generalization issue regarding these parameters. On the other hand, the RBF model excels in predicting COND−D but struggles with the other parameters. Meanwhile, the MLP-PCA model significantly struggles across all parameters, with R values ranging from 0.315 to 0.795, indicating the model's inability to capture complex relationships between input data and outputs.

**Table 6:** The performance of the proposed models during the training phase: Second step.

Matrices/Parameters	PH-D	BOD-D	COD-D	SS-D	SSV-D	SED-D	COND-D
MLP−PCA							
NRMSE	0.096	0.112	0.127	0.124	0.121	0.112	0.109
NMAE	0.075	0.083	0.102	0.086	0.092	0.069	0.083
R	0.795	0.650	0.639	0.358	0.469	0.315	0.317
GFFR							
NRMSE	0.095	0.110	0.115	0.118	**0.095**	0.111	0.039
NMAE	0.075	0.085	0.092	**0.082**	**0.072**	**0.065**	0.028
R	0.805	0.680	**0.728**	0.469	**0.725**	0.365	**0.947**
MLP							
NRMSE	**0.093**	**0.105**	**0.114**	**0.115**	0.096	0.110	**0.037**
NMAE	**0.072**	**0.079**	**0.091**	0.084	0.074	0.068	**0.024**
R	**0.810**	**0.694**	0.725	**0.496**	0.714	0.364	0.945
RBF							
NRMSE	0.099	0.109	0.117	0.116	0.099	**0.107**	0.043
NMAE	0.077	0.082	0.093	0.084	0.076	0.068	0.030
R	0.779	0.664	0.707	0.487	0.692	**0.418**	0.928

**Table 7:** The performance of the proposed models during the testing phase: Second step.

Matrices/Parameters	PH-D	BOD-D	COD-D	SS-D	SSV-D	SED-D	COND-D
MLP−PCA							
NRMSE	0.097	0.091	0.132	0.112	0.130	**0.089**	0.096
NMAE	0.077	**0.069**	0.097	0.085	0.100	**0.065**	0.075
R	0.870	0.779	0.657	0.374	0.398	0.292	0.506
GFFR							
NRMSE	**0.093**	0.105	0.133	0.118	0.102	0.093	0.048
NMAE	**0.074**	0.081	0.100	0.084	**0.079**	0.066	0.032
R	**0.875**	0.711	0.661	0.342	**0.683**	0.202	**0.915**
MLP							
NRMSE	0.097	**0.089**	**0.128**	**0.108**	**0.101**	0.091	**0.046**
NMAE	0.077	0.070	0.096	**0.080**	**0.079**	**0.065**	**0.028**
R	0.866	**0.784**	**0.692**	**0.460**	0.678	**0.297**	0.910
RBF							
NRMSE	0.104	0.092	0.128	0.113	0.104	0.093	0.056
NMAE	0.081	0.070	**0.094**	0.083	0.083	0.069	0.036
R	0.850	0.762	0.688	0.362	0.659	0.239	0.863

During the testing phase, both MLP and GFFR models continue to demonstrate robustness for COND−D prediction with R=0.91, NRMSE=0.046, and NMAE=0.028, and R=0.915, NRMSE=0.048, and NMAE=0.032 indicating the models’ ability to explain the majority of variance in the data with minor errors. For PH−D prediction, the GFFR model slightly outperforms the MLP model with R=0.875, NRMSE=0.093, and NMAE=0.074, although both models maintained a moderate performance with the GFFR having slight edge improvement in handling PH values. Meanwhile, the RBF model showed an excellent performance in predicting COND−D, where the model can explain about 92.8 % of the variance with minor errors.

However, all model struggles significantly regarding (BOD−D), (COD−D), (SS−D), (SSV−D), and (SED−D) prediction with R values below 0.728, indicating generalization and reliability issues.

Moving to the final step of this scenario, which determines the general performance of this scenario. This scenario involves using the models from the previous step to generate new values for the parameters (PH−D,COD−D,SS−D,SSV−D,SED−D,andCOND−D) and using them as inputs along with (Q−E) to develop new models taking into account the ${\text{WWTP}}^{\prime }$ s outputs in the developing process. The performance of the proposed models is presented in Table 8, Table 9 for the training and testing phase, respectively.

**Table 8:** The performance of the proposed models during the training phase: Final step.

Matrices/Parameters	PH-S	BOD-S	COD-S	SS-S	SSV-S	SED-S	COND-S
MLP−PCA							
NRMSE	0.074	0.068	0.118	0.081	0.123	0.070	0.157
NMAE	0.049	0.025	0.074	**0.040**	0.093	0.017	0.121
R	0.253	0.142	0.277	0.136	0.278	0.040	0.426
GFFR							
NRMSE	0.076	**0.066**	0.119	0.080	0.117	0.069	0.096
NMAE	0.052	0.032	0.082	0.047	0.091	0.019	0.071
R	**0.378**	0.283	**0.315**	0.242	0.433	0.129	**0.879**
MLP							
NRMSE	0.072	**0.064**	0.117	**0.077**	0.118	**0.066**	**0.091**
NMAE	**0.046**	**0.025**	0.074	0.042	0.089	**0.017**	**0.064**
R	0.341	**0.375**	0.312	**0.350**	0.401	**0.351**	0.854
RBF							
NRMSE	**0.071**	0.066	**0.117**	0.080	**0.114**	0.069	0.091
NMAE	0.047	0.026	**0.073**	0.042	**0.085**	0.019	0.064
R	0.380	0.260	0.313	0.212	**0.464**	0.165	0.852

**Table 9:** The performance of the proposed models during the testing phase: Final step.

Matrices/Parameters	PH-S	BOD-S	COD-S	SS-S	SSV-S	SED-S	COND-S
MLP−PCA							
NRMSE	0.068	**0.021**	0.085	**0.041**	0.134	0.017	0.162
NMAE	0.052	**0.017**	0.068	**0.032**	0.102	0.012	0.120
R	0.420	**0.268**	0.356	**0.277**	0.025	0.048	0.462
GFFR							
NRMSE	0.071	0.038	0.090	0.056	**0.124**	**0.020**	**0.091**
NMAE	0.056	0.028	0.076	0.045	**0.096**	**0.014**	**0.071**
R	0.493	0.176	0.368	−0.110	**0.270**	**0.159**	**0.893**
MLP							
NRMSE	**0.064**	0.025	**0.082**	0.047	0.138	0.023	0.089
NMAE	**0.051**	0.019	**0.064**	0.036	0.103	0.012	0.065
R	**0.491**	0.218	**0.433**	0.102	0.105	0.014	0.875
RBF							
NRMSE	0.070	0.026	0.084	0.042	0.133	0.020	0.088
NMAE	0.054	0.020	0.065	0.033	0.100	0.013	0.062
R	0.313	0.216	0.373	0.254	0.175	0.119	0.878

During the training phase, all models showed varied performance in predicting all water quality parameters. The MLP, GFFR, and RBF models give a moderate prediction regarding COND−S, reaching the best prediction with MLP model with R=0.854, NRMSE=0.064, and NMAE=0.091, showing the model capacity and consistent strength to predict conductivity. However, all models struggle significantly regarding the other parameters with R values below 0.5, indicating that the models can explain less than 50 % of the variance in data and the inability to capture complex relationships.

**Fig. 5:**
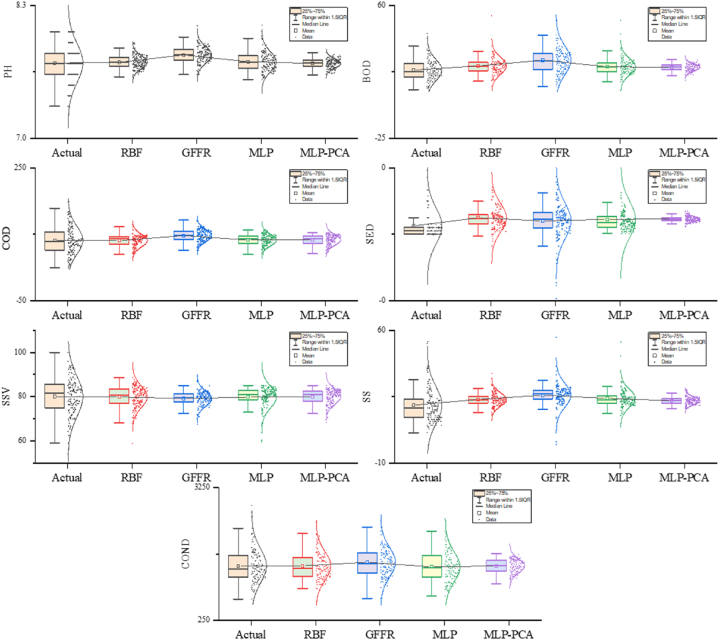
Box plot showing the performance of the proposed models during the testing phase: Final step.

Table 9 shows the performance of the proposed models during the testing phase, showing that both MLP, RBF, and GFFR performed slightly better performance than in the training phase regarding COND−S prediction, reaching the best performance with the GFFR model with an R-value of 0.893, followed by the RBF model with an R-value of 0.878, indicating the models' ability to explain the variance by 87.5 %–89.3 %. Although all models showed better performance in the testing phase compared to training, all models struggled significantly in predicting the other parameters with R values below 0.5, indicating the models' inability to explain more than 50 % of the variance in data. Fig. 5 shows that MLP, RBF, and GFFR models closely match predicted values regarding COND−S with medians that are closely aligned. Moreover, the spread of the interquartile range (IQR) is minimal, indicating the strong performance and reliability of models in predicting conductivity. Meanwhile, for the other parameters, the box plot shows that all models suffer from significant deviations between actual and predicted values. Furthermore, the medians of these models are far from the actual ones with large IQR values and outliers, suggesting that models fail to capture complex relationships and patterns.

The proposed modes in scenarios 1 and 2 show a significantly low prediction capacity for water quality parameters. Moreover, no dominant model can provide efficient predictions for all water quality parameters since each scenario has its specific best model for specific parameter prediction. Nevertheless, the approaching process of reaching the final predictions is significantly different in terms of improving the prediction capacity of the proposed models. In this regard, Table 10 shows the best predictive model for each parameter as well as the improvement in the prediction capacity (in terms of R) induced by Scenario 2 compared to Scenario 1. According to Table 10, the second scenario significantly increased the prediction capacity of the proposed models, with increments ranging from 0.2 % to 157 % and an average of 60 %.

**Table 10:** The best predictive model for each parameter in S1 and S2.

	Scenario 1 (S1)				Scenario 2 (S2)				Improvement by S2
	MLP−PCA	GFFR	MLP	RBF	MLP−PCA	GFFR	MLP	RBF	NRMSE%
PH	**✓**						**✓**		73
BOD				**✓**	**✓**				75
COD			**✓**				**✓**		24
SS				**✓**	**✓**				156
SSV		**✓**				**✓**			34
SED			**✓**			**✓**			61
COND		**✓**				**✓**			0.2

## 5 Conclusion

The research is conducted to come up with an elaborate comparative study of four machine learning models (MLP, GFFR, MLP-PCA, RBF) in relation to predicting key water quality parameters in wastewater treatment plants (WWTPs). In this regard, two distinct scenarios have been proposed: single-step modeling, which incorporates exclusively WWTP inputs and outputs, and multi-step modeling, which incorporates outputs from primary and secondary settlers to reach the final prediction. Furthermore, this study also investigates the efficiency of the adopted model against high-dimensional data induced by the second scenario as a result of more data points and outputs being progressively integrated at each step.

For the first scenario, all models struggle to predict most of the water quality parameters except conductivity (COND), where the GFFR model shows an exceptional performance in which the model explains 89.7 % of the variance of data along with minor prediction deviations, followed by the MLP model with 83.6 % of the variance is explained by the model. Regarding the other parameters, all models lag significantly in their performance, with low correlation and high deviations between actual and predicted values, reflecting the models' limitations in capturing complex relationships and patterns. This can be interpreted as this scenario oversimplifying the process of WWTP by relying on single-step prediction without considering the output from primary and secondary settlers. Consequently, the models miss critical information about transformations that happen in the primary and secondary settlers, limiting the presentation of the full spectrum of variations within the process, which in turn leads to significant deviations and outliers in the prediction process.

For the second scenario, all models performed significantly poor in predicting most of the water quality parameters, except conductivity (COND), where the GFFR model ranked the best performance with an R-value of 0.893, followed by RBF model with an R-value of 0.878, and finally the MLP model with an R-value of 0.875. Furthermore, all models showed significantly improved performance in the second scenario compared to the first scenario, with an improvement rate between 0.2 % and 157 % and an average of 60 %. This is due to the incorporation of intermediate data from primary and secondary settlers, providing valuable information about the transformation that occurred in the WWTP. However, despite these improvements, all models failed to provide uniform and robust predictions for most water quality parameters, resulting in a significantly poor performance across almost all parameters. This can be interpreted as the introduction of high dimensionality into the dataset as a result of using models to generate new data and utilizing them to build subsequent models, posing significant challenges in terms of the complexity of data and models' generalization ability. As a result, this study suggests using hybrid models with larger data set that combine the strengths of multiple models, enabling the models to be more efficient in capturing complex relationships and high dimensionality in data. Furthermore, this study also suggests using sequential modeling techniques that utilize the outputs of one model as inputs for another model, enabling the models to capture more complex relationships and patterns by breaking the prediction process into smaller and manageable parts. Moreover, advanced dimensionality techniques are recommended to be utilized, and more real-time data is integrated along with feature selection optimizers.

## CRediT authorship contribution statement

**Haitham Abdulmohsin Afan:** Writing – original draft, Methodology, Formal analysis, Conceptualization. **Wan Hanna Melini Wan Mohtar:** Validation, Methodology, Investigation. **Faidhalrahman Khaleel:** Writing – original draft, Investigation, Formal analysis. **Ammar Hatem Kamel:** Visualization, Resources, Methodology. **Saif Saad Mansoor:** Visualization, Software, Data curation. **Riyadh Alsultani:** Software, Formal analysis, Data curation. **Ali Najah Ahmed:** Supervision, Investigation, Conceptualization. **Mohsen Sherif:** Supervision, Methodology, Conceptualization. **Ahmed El-Shafie:** Writing – review & editing, Validation, Supervision.

## Declaration of competing interest

The authors declare that they have no known competing financial interests or personal relationships that could have appeared to influence the work reported in this paper.
